# Merged swing-muscle synergies and their relation to walking characteristics in subacute post-stroke patients: An observational study

**DOI:** 10.1371/journal.pone.0263613

**Published:** 2022-02-04

**Authors:** Naomichi Mizuta, Naruhito Hasui, Yuki Nishi, Yasutaka Higa, Ayaka Matsunaga, Junji Deguchi, Yasutada Yamamoto, Tomoki Nakatani, Junji Taguchi, Shu Morioka

**Affiliations:** 1 Department of Neurorehabilitation, Graduate School of Health Sciences, Kio University, Koryo, Japan; 2 Department of Therapy, Takarazuka Rehabilitation Hospital (SHOWAKAI Medical Corporation), Takarazuka, Japan; 3 Department of Rehabilitation, Nakazuyagi Hospital (HIMAWARIKAI Medical Corporation), Tokushima, Japan; 4 Neurorehabilitation Research Center, Kio University, Koryo, Japan; Toronto Rehabilitation Institute - UHN, CANADA

## Abstract

In post-stroke patients, muscle synergy (the coordination of motor modules during walking) is impaired. In some patients, the muscle synergy termed module 1 (hip/knee extensors) is merged with module 2 (ankle plantar flexors), and in other cases, module 1 is merged with module 4 (knee flexors). However, post-stroke individuals with a merging pattern of module 3 (hip flexor and ankle dorsiflexor) and module 4, which is the swing-muscle synergy, have not been reported. This study aimed to determine the muscle-synergy merging subtypes of post-stroke during comfortable walking speed (cws). We also examined the effect of experimental lower-limb angle modulation on the muscle synergy patterns of walking in each subtype. Forty-one participants were assessed under three conditions: cws, long stepping on the paretic side (p-long), and long stepping on the non-paretic side (np-long). Lower-limb flexion and extension angles and the electromyogram were measured during walking. Subtype classification was based on the merging pattern of the muscle synergies, and we examined the effect of different lower-limb angles on the muscle synergies. We identified three merging subtypes: module 1 with module 2 (subtype 1), module 1 with module 4 (subtype 2), and module 3 with module 4 (subtype 3). In the cws condition, the lower-limb flexion angle was reduced in subtype 3, and the lower-limb extension angle was decreased in subtype 1. A more complex muscle synergy was observed only in subtype 3 in the p-long condition versus cws (*p* = 0.036). This subtype classification of walking impairments based on the merging pattern of the muscle synergies could be useful for the selection of a rehabilitation strategy according to the individual’s particular neurological condition. Rehabilitation with increased lower-limb flexion may be effective for the training of patients with merging of modules 3 and 4 in comfortable walking.

## 1. Introduction

Gait asymmetry is a characteristic feature of stroke-related walking disorder and is associated with increased the risk of falls [1, 2]. Recent studies suggest that the level of asymmetry is more important than deficits in walking speed for understanding the degree of walking deficit and the compensatory mechanisms in post-stroke patients [3–6]. Many post-stroke patients are observed to have asymmetric flexion or extension angles of the lower-limb during comfortable walking [5, 7]; however, the characteristics of this asymmetry differ across individual patients, who may exhibit either a reduced flexion or extension angle of the paretic lower-limb [8, 9]. Gait asymmetry is related to impaired timing of lower-limb muscle activity [10]. Therefore, the abnormal patterns of lower-limb muscle activity observed during various phases of the stance or swing phase are considered to influence the differences in asymmetry characteristics.

In healthy subjects, muscle activity during comfortable walking is composed of four independent muscle synergies: hip and knee extensors (module 1), ankle plantar flexors (module 2), hip flexors and ankle dorsiflexors (module 3), and knee flexors (module 4) [11]. For the synergy analysis, evaluating the "total variance accounted for" (VAF) by a given number of synergies allows to quantify the complexity of an individual’s muscle activation pattern [12–20]. The VAF by one synergy (VAF1) is a summarized measure of synergy complexity. When VAF1 is high, one synergy can explain most of the variance in muscle activation, which indicates a reduction in the complexity of motor control during the task [12, 16–20].

In healthy subjects, the mathematical weight coefficients assigned to the muscles to model each synergy do not change significantly when walking speed changes [21, 22]. However, many chronic post-stroke patients show a merging of these muscle synergies during comfortable walking [13–15, 23–25]. Walking subtypes have been reported to show a merging of module 1 with module 2 or module 1 with module 4 in chronic post-stroke patients [13, 26]. Thus, muscle synergy deficits due to stroke are categorized based on the merging patterns. Walking disorders such as decreased step length on the paretic side and abnormal knee movement during the swing phase are observed in chronic post-stroke patients [5, 27]. We believe that some patients have a merging pattern of module 3 with module 4, i.e., impaired swing muscle synergy, underlying such abnormal movement patterns during the swing phase. However, the identification and walking characteristics of this subtype have not been previously described. Moreover, most previous studies examining muscle synergy during walking following a stroke have involved patients in the chronic phase [13, 15, 23–25], and the characteristics of the subtype in subacute patients are unclear. Subtype classification based on the pathogenesis of the walking deficit is important for providing optimal rehabilitation.

The merging of muscle synergies is related to lower-limb angle and asymmetry in post-stroke patients, and is an important variable for understanding both the pathological neural mechanisms and the kinematics [24, 28]. However, because previous studies did not experimentally modify the movement of the paretic lower-limb [24, 28], the causal relationship between muscle synergy complexity and lower-limb angles while walking has not yet been fully elucidated. Therefore, it is necessary to design experiments that modify the lower-limb angle during walking in post-stroke patients and that can prove a causal relationship of this variable with muscle synergy. Furthermore, the effects of experimentally altering the lower-limb angle during walking on muscle synergy complexity are expected to be specific to each merging subtype. This is because we expect that each subtype will exhibit a particular decrease in flexion or extension angle of the lower-limb during comfortable walking [8, 9]. If the forward stride of the paretic side is reduced during comfortable walking, the passive knee flexion mechanism does not function properly [29]. This likely causes early activation of module 4 during the swing phase, leading to increased foot clearance, while decreasing the forward stride length on the paretic side. Therefore, in the subtype where modules 3 and 4 are merged during comfortable walking, walking with increased lower-limb flexion angle during the swing phase may complicate muscle synergy. Identifying the effects of experimental modulation of these angles on muscle synergy for each merging subtype will enable walking rehabilitation based on the particular neurological deficits of each patient.

We anticipate that the subtype in which module 3 and module 4 are merged will show decreased lower-limb flexion angle during comfortable walking because of impairment in the swing phase on the paretic side. We hypothesized that the experimental modification of the lower-limb flexion or extension angles during walking affects the complexity of the muscle synergy to a degree that depends on the merging pattern. Moreover, we hypothesized that the 3–4 merged subtype represents a complex muscle synergy by experimentally increasing the lower-limb flexion angle on the paretic side.

Based on the observed muscle-synergy merging patterns, we identified three pathological walking subtypes. We examined the effect of experimental modification of the lower-limb flexion/extension angles on the muscle synergy patterns for each subtype in subacute post-stroke patients. These results can contribute to the development of effective, individualized intervention strategies for walking rehabilitation in post-stroke patients.

## 2. Materials and methods

### 2.1. Participants

In this cross-sectional study, 41 post-stroke patients (mean ± standard deviation, 72.3 ± 7.50 years; stroke onset, 75.0 ± 34.5 days) were enrolled at the Takarazuka Rehabilitation Hospital of SHOWAKAI Medical Corporation and the Nakazuyagi Hospital of HIMAWARIKAI Medical Corporation. This study was conducted according to the Strengthening the Reporting of Observational Studies in Epidemiology (STROBE) checklist. The exclusion criteria were: (1) inability to walk independently without the assistance of physical therapists, (2) inability to walk without using walking aids, (3) presence of bilateral lesions, (4) a Mini-Mental State Examination score <24 points, (5) a history of orthopedic disease, (6) presence of pain, (7) presence of cerebellar lesions or resting tremor, and (8) presence of unilateral spatial neglect, except in the context of stroke. Patients who did not meet any of the exclusion criteria were enrolled. All participants provided informed consent before the start of the study. All procedures were approved by the ethics committee of Takarazuka Rehabilitation Hospital of Medical Corporation SHOWAKAI (ethics review number: 2019-P-2) and were conducted in accordance with the Declaration of Helsinki.

### 2.2. Experimental set-up and procedures

Participants were instructed to walk five times on a 10-m walkway with a supplementary 6-m walkway, while assisted by a nearby physical therapist to eliminate the risk of falling. The participants were allowed to use a cane as necessary during assessments; however, lower-limb orthotics were not allowed. During walking, video recording was performed (sampling rate: 60 Hz) and foot-pressure data from a wireless insole sensor (physical information therapy, Reif Co., Ltd., Japan; sampling rate: 100 Hz), data from an acceleration sensor, and electromyographic recordings were acquired (Delsys Trigno, Delsys Inc., Boston, MA; sampling rate: 1926 Hz). The video camera recorded from the sagittal plane during walking, and the plantar pressure recorded from both legs. Wireless surface electromyography was recorded on the paretic side from the tibialis anterior, soleus, medial gastrocnemius, vastus medialis, rectus femoris, semitendinosus, biceps femoris, and gluteus medius [30]. The wireless electromyographic montage was equipped with a 3-axis accelerometer. Each skin site was shaved and cleaned with alcohol prior to electrode placement. Before recording, we confirmed a score of <4 on the modified Borg scale to ensure that fatigue did not affect performance.

### 2.3. Clinical evaluation

The Fugl–Meyer assessment (FMA) was used to measure the severity of motor paralysis and sensory disturbance. The FMA synergy score was used to determine the FMA motor score [31]. To evaluate spasticity, we assessed the responses of the ankle plantar flexor muscle using a modified Ashworth Scale converted to a 0–5-point scale (0: no increase in muscle tone, 5: the affected part is rigid in flexion or extension). We also assessed motor performance using the Trunk Impairment Scale, the short-form Berg Balance Scale, and the Functional Ambulation Category.

### 2.4. Data recording and analysis

Participants walked under three conditions: comfortable walking speed (cws), long stepping on the paretic side (p-long), and long stepping on the non-paretic side (np-long). In the cws condition, participants were instructed to walk at a comfortable speed. In the p-long condition, they were instructed to walk with a large forward swing of the paretic lower-limb. In the np-long condition, they were instructed to walk with a large forward swing of the non-paretic lower-limb. The choice of manipulating the lower-limb angle in the p-long and np-long conditions was based on previous findings that neural-system control of limb kinematic parameters is more fundamental to walking than is control of joint kinematic parameters [32, 33]. The measurement order of the three walking conditions was randomized (randomized block design). Under all walking conditions, participants were instructed to walk at a comfortable speed, and the subjects practiced each condition for 5 min to familiarize with the procedure prior to data collection. Using the recorded video data, walking speed and cadence were measured using a stopwatch when participants crossed the start and end lines of the 10-m walkway. To prevent any confounding effects due to acceleration and deceleration, the first and last three gait cycles were removed from the dataset. Twenty strides were then extracted from each walking session [34]. The symmetry of the single-leg support time was evaluated using the Symmetry Index [35]. To calculate joint angle, the coordinates of the hip and ankle joint centers were obtained using OpenPose (version 1.4.0, https://github.com/CMU-Perceptual-Computing-Lab/openpose) to analyze the recorded video [36]. OpenPose is a markerless motion capture system that estimates posture from video camera images, and its reliability has been confirmed by optical motion capture [37]. The lower-limb angle on the paretic side was defined as the angle between the vertical axis and the vector joining the hip and ankle joints [38]. The direction of flexion was defined as positive. The angle data was low-pass filtered with a cutoff of 6 Hz using a zero-lag 4^th^-order Butterworth filter [15]. The outcome “peak angle during walking” was based on the lower-limb flexion and extension angles on the paretic side. The raw electromyogram (EMG) signals were band-pass filtered with cutoff frequencies in the range of 20–500 Hz using a zero-lag 4^th^-order Butterworth filter, after which they were de-meaned, rectified, and low-pass filtered using a zero-lag 4^th^-order Butterworth filter with a cutoff frequency of 10 Hz. The EMG was normalized by dividing by the maximum amplitude recorded [39]. All pre-processing EMG procedures were performed according to the guidelines for Surface Electromyography for the Non-Invasive Assessment of Muscles (http://www.seniam.org). The walking events were identified from the anterior-posterior component of the tri-axial accelerometer attached to the shank on the paretic side and the wireless insole foot pressure sensors on both sides [40]. In addition, to identify walking events from joint angles, the ankle joint coordinate data were differentiated twice to convert them to acceleration data, after which they were used to determine heel contact times for both legs. Within each gait cycle, shank acceleration, insole foot pressure, and joint angle data were linearly interpolated to normalized times in a 100-point walking-cycle time base.

For each walking condition, non-negative matrix factorization (NNMF) was used to extract muscle synergies from the concatenated EMG data [13]. The EMG data were time-normalized by the gait cycle period before calculation of muscle synergy. The NNMF was performed using a multiplicative update algorithm. The NNMF parameters were as follows: tolerance for the residual = 1e^-6^; tolerance for the relative change in elements = 1e^-4^. The algorithm was repeated 1,000 times and the result with the lowest root-mean-square residual was used [41, 42]. Synergies were allowed to vary in each condition. NNMF decomposes the EMG signals into two matrices: *W*, containing the synergy weights, which are the weighted contributions of each included muscle to each synergy, and *C*, the synergy activations, such that:

$$EMG=(W_{m*n}*C_{n*t})+error$$ (1)

In Eq (1), *n* is the number of synergies, *m* is the number of muscles, and *t* is the number of data points. The *error* value is the difference between the measured EMG data and EMG signals reconstructed from the calculated synergy. *error* was used to calculate the VAF as:

$$VAF_{n}=\left(1-\frac{\left[\sum_{j}^{t}\sum_{j}^{m}{\left(error\right)}^{2}\right]}{\left[\sum_{j}^{t}\sum_{j}^{m}{\left(EMG\right)}^{2}\right]}\right)$$ (2)

The number of synergies selected for use in further analysis was the highest in which the VAF by the reconstructed EMG envelope was above 90%, and a further increase in the number of synergies did not yield more than a 5% increase in the VAF [13, 41]. We also added a local criterion whereby muscle synergy accounted for more than 75% of the variability in each muscle [43, 44]. Additionally, the VAF by one synergy was used as an additional estimate of synergy complexity [16, 17, 45]. MATLAB R2017a (MathWorks, Inc., Natick, MA, USA) was used for all data analyses.

### 2.5. Statistical analysis

Because the analysis focused on the merging patterns of muscle synergies, only participants with three muscle synergies were included in the analysis. To characterize the merging pattern, we first determined whether a given muscle significantly contributed to a given synergy. This was defined as a corresponding muscle weight coefficient of at least 0.3 in the cws condition [46, 47]. The merging-pattern subtypes were identified by comparing these weight coefficients across synergies. A Shapiro–Wilk test was used to assess the assumption of normality. To compare the general characteristics, clinical evaluation, and walking variables across subtypes, a one-way analysis of variance (ANOVA) or the Kruskal–Wallis and Steel–Dwass tests were used. In the cws condition, to compare lower-limb flexion and extension angles across subtypes, we used an ANOVA with three subtypes (i.e., subtypes 1, 2, and 3). To assess the effect of the walking condition in all participants on walking speed, cadence, symmetry index, lower-limb peak flexion and extension angles, and VAF1 a repeated-measures one-way analysis of variance was performed. To confirm the effect of the walking condition among subtypes, a 3 × 3 [Condition (cws/p-long/np-long) × Subtype (1/2/3)] repeated-measures two-way analysis of variance (RM-ANOVA) was conducted to examine the lower-limb flexion and extension angles and VAF1 during walking. When Mauchly’s sphericity test was significant, the repeated-measures one-way analysis of variance and RM-ANOVA were corrected by Greenhouse-Geisser if necessary. All post-hoc tests were followed by Bonferroni post-hoc testing. Partial eta squared (η_p_^2^) values were calculated as a measure of effect size. A common interpretation is to categorize the effect size into small (η_p_^2^  =  0.01), medium (η_p_^2^  =  0.06), and large (η_p_^2^  =  0.14) [48]. To determine the association between VAF1 and walking speed, we used Spearman’s rank correlation analysis. All analyses were completed using R statistical software (Ver.4.0.5, R Core Team, 2021). Statistical significance was assumed at *p* < 0.05, and values are reported as means and standard errors.

## 3. Results

### 3.1. Muscle-synergy merging patterns: Subtype classification

**Fig 1:**
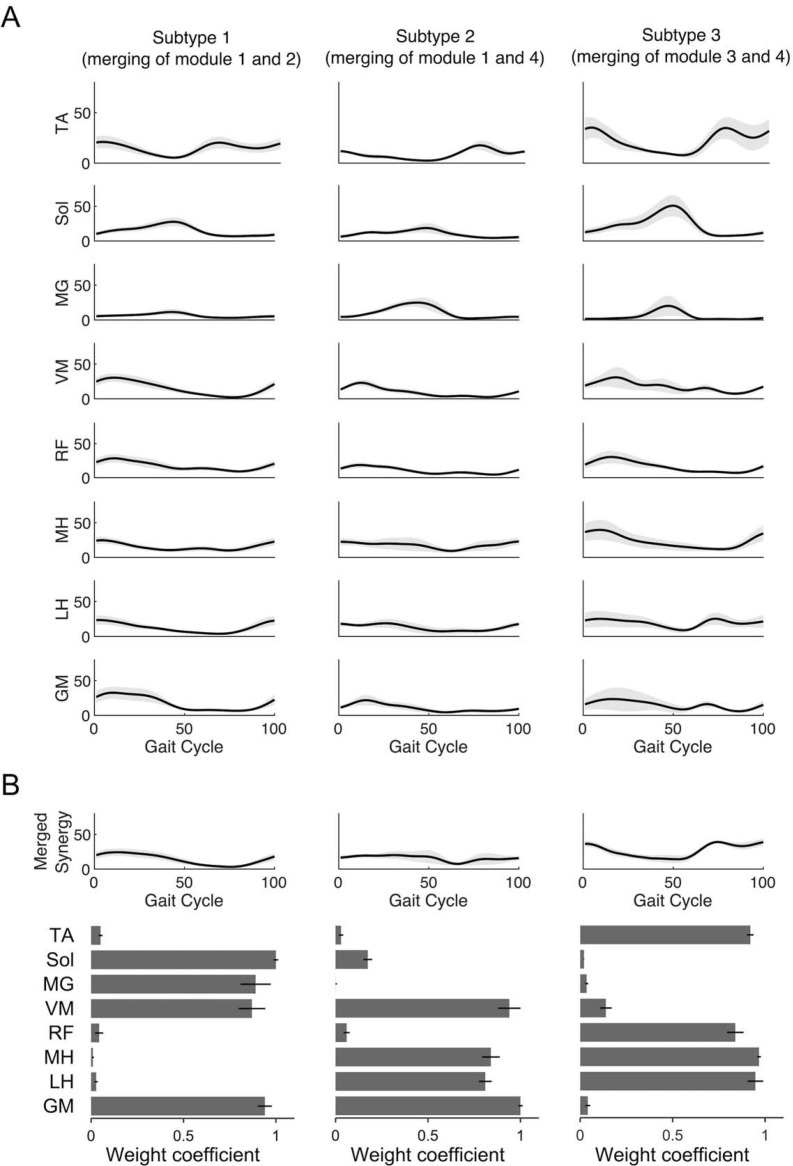
Muscle activity for each subtype during walking. (A) Solid black lines and gray areas represent the mean ± 95% confidence interval. The values are given as percentage. From the top to bottom the figure shows the tibialis anterior (TA), soleus (Sol), medial gastrocnemius (MG), vastus medialis (VM), rectus femoris (RF), semitendinosus (MH), biceps femoris (LH), and gluteus medius (GM) muscle activity during the 100 percent gait cycle in comfortable walking speed. (B) The data are reported as the mean ± 95% confidence interval. The graph indicate the subtypes in the merging pattern of muscle synergy in comfortable walking speed, where subtype 1 is the merging pattern of modules 1 and 2, subtype 2 is the merging pattern of modules 1 and 4, and subtype 3 is the merging pattern of modules 3 and 4. The bottom bar graph shows the weight coefficients for the eight muscles on the paretic side included in the merged muscle synergy.

The pattern of muscle activity during comfortable walking differed across participants; some cases showed an increase or decrease in muscle activity with gait-cycle phase, while others showed little change. Especially in subtype 3, we observed that the activation of the tibialis anterior was greater than that of the other subtypes during the swing phase, and that the lateral hamstrings was activated early in the swing phase (see Fig 1A). Because we focused on investigating the characteristics of the merging pattern of muscle synergies during walking, we restricted our study to participants with three muscle synergy counts identified based on the VAF for the comfortable walking speed (cws) condition. Participant-specific examination of the structure of the merged synergies confirmed the presence of various patterns, without any dominant pattern. Three merging subtypes were identified in the comfortable walking data (see Fig 1B): merging of modules 1 and 2 (subtype 1, n = 12), merging of modules 1 and 4 (subtype 2, n = 13), and merging of modules 3 and 4 (subtype 3, n = 12). Although the present study was limited to participants with three muscle synergies based on total VAF, the merging pattern revealed three subtypes (see Table 1). The merging of muscle synergies in each subtype was observed to be characterized during the stance phase for subtype 1, during the transition from swing to stance for subtype 2, and during the swing phase for subtype 3.

**Table 1:** Characteristics of each subtype.

	Subtype 1 (n = 12)	Subtype 2 (n = 13)	Subtype 3 (n = 12)	*p* value
Age (years)	69.8 ± 8.07	71.2 ± 7.61	75.6 ± 6.39	0.15
Sex (n): male / female	11 / 1	11 / 2	6 / 6	-
Affected side (n): right / left	4 / 8	9 / 4	9 / 3	-
Time since stroke (days)	81.8 ± 37.0	78.3 ± 43.4	65.1 ± 21.8	0.48
Functional Ambulation Category	3.50 ± 0.67	3.27 ± 0.47	3.25 ± 0.45	0.57
Using assist device (n): no use / T-cane / Q-cane	6 / 6 / 0	9 / 4 / 0	12 / 0 / 0	-
FMS (lower extremity): max = 22	19.8 ± 3.71	20.5 ± 2.62	22.0 ± 0.00	< 0.01^a^
FMA sensory score (lower extremity): max = 12	10.0 ± 2.41	10.5 ± 1.64	9.83 ± 1.99	0.71
Modified Ashworth Scale: max = 5*	0.67 ± 0.99	0.64 ± 1.12	0.25 ± 0.45	0.71
Trunk Impairment Scale	16.0 ± 5.48	19.1 ± 2.62	18.6 ± 3.13	0.20
Short Form Berg Balance Scale	22.4 ± 5.09	22.0 ± 3.13	20.2 ± 1.39	0.40
Waking speed (m / s)	0.79 ± 0.37	0.86 ± 0.21	0.85 ± 0.12	0.78
Cadence (step / min)	100.9 ± 25.5	104.6 ± 16.0	107.1 ± 8.34	0.70
Symmetry index in single support phase (%)^†^	-4.25 ± 4.99	-2.53 ± 2.02	-1.98 ± 2.01	0.23
Lower-limb flexion angle (deg)^‡^	14.5 ± 3.37	14.5 ± 1.97	10.2 ± 1.89	< 0.01^b^
Lower-limb extension angle (deg)^‡^	-14.4 ± 3.81	-19.4 ± 3.75	-20.5 ± 4.42	< 0.01^c^

The data are reported as the mean or n ± standard deviation. The *p* value indicates the results of the one-way analysis of variance or Kruskal-Wallis test among subtypes. The walking parameters correspond to the results of the cws condition.

*) To evaluate the spasticity of the ankle plantar flexor muscle, a Modified Ashworth Scale was used and evaluated on a 0–5 scale

†) If the symmetry index was low, the paretic side in the single-leg support phase was shorter than the non-paretic side.

‡) The higher value was set as the direction of flexion

a) Subtype 3 was higher than subtypes 1 and 2 using the Kruskal-Wallis test (Steel-Dwass for post-hoc test).

b) Subtype 3 was lower than subtypes 1 and 2 using the one-way analysis of variance test (Bonferroni correction for post-hoc test).

c) Subtype 1 was higher than subtypes 2 and 3 using the one-way analysis of variance test (Bonferroni correction for post-hoc test).

Abbreviations: FMS, Synergy score of the Fugl–Meyer assessment; FMA, Sensory score of the Fugl–Meyer assessment.

### 3.2. Kinematic characteristics by subtype

Because the merging pattern of muscle synergies was characterized into three subtypes, we first examined the specific walking characteristics of each subtype in the cws condition. In the cws condition, lower-limb flexion and extension angles depended on subtype. In this condition, the lower-limb flexion angle was smaller in subtype 3 than in subtype 1 (10.2 ± 0.55 versus 14.5 ± 0.97, *p* = 0.02) or subtype 2 (10.2 ± 0.55 versus 14.5 ± 0.57, *p* = 0.02, Table 1). In contrast, the lower-limb extension angle in the cws condition was smaller in subtype 1 than in subtype 3 (-14.4 ± 1.10 versus -20.5 ± 1.28, *p* = 0.002, Table 1). Therefore, in the cws condition, subtype 1 with merging stance phase muscle synergy decreased the lower-limb extension angle, and subtype 3 with merging swing phase muscle synergy reduced the lower-limb flexion angle.

**Fig 2:**
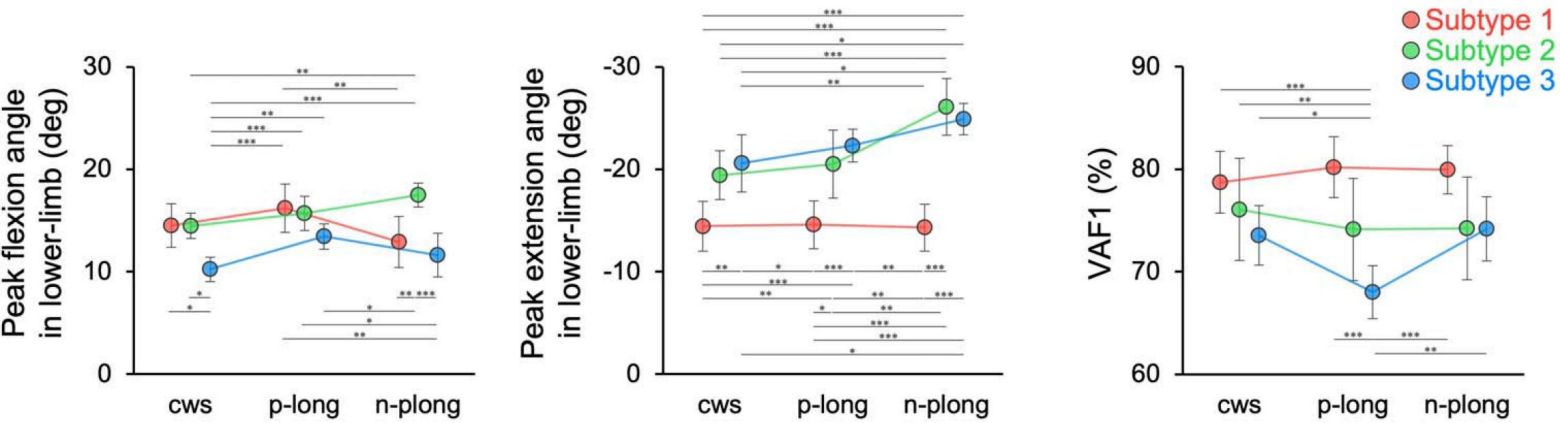
Comparison of walking parameters and VAF1 in each walking condition. The data are reported as the mean ± 95% confidence interval. The peak flexion angle of the lower-limb, the peak extension angle of the lower-limb, and the VAF1 are shown from left to right. The colors indicate subtype differences and represent these variables across walking conditions (i.e., cws, p-long, and np-long conditions). The peak flexion and extension angles of the lower-limb differed among gait conditions for subtypes 2 and 3. VAF1 of subtype 3 had a reduced p-long compared to the cws condition. On the other hand, VAF1 in subtypes 1 and 2 did not differ in each walking condition. *p < 0.05, **p < 0.01, ***p < 0.001. Abbreviations: cws, comfortable walking speed; p-long, paralytic side long step; np-long, non-paralytic side long step; VAF, variance-accounted-for.

Next, the cws, p-long, and np-long conditions were used to examine the effects of lower-limb flexion and extension angles on muscle synergy during walking. The validity of these walking conditions was confirmed by the difference between the walking speed, and lower-limb flexion and extension angles. Averaging across all participants, walking speed (*p* = 0.24) and symmetry index in single support phase (*p* = 0.21, see Table 2) did not depend on walking condition; however, the cadence was lower in the cws condition than in the p-long (104.9 ± 2.95 versus 98.2 ± 3.10, *p* = 0.002) and np-long (98.8 ± 2.95, *p* = 0.013) conditions. Across all participants, the lower-limb flexion angle was increased in the p-long condition compared to the cws condition (15.1 ± 0.53 versus 13.1 ± 0.53, *p* < 0.001, Table 2), and the lower-limb extension angle in the np-long condition was greater than in the cws condition (-21.8 ± 1.06 versus -18.1 ± 0.79, *p* < 0.001) or p-long condition (-21.8 ± 1.06 versus -19.1 ± 0.85, *p* = 0.006, Table 2). Because the lower-limb flexion angle was increased in the p-long condition and the lower-limb extension angle was increased in the np-long condition, these walking conditions allowed the experimental manipulation of the kinematic parameters. However, lower-limb flexion and extension angles differed across walking conditions in each subtype (see Fig 2).

**Table 2:** Spatio-temporal parameters between walking conditions in all participants.

	Walking conditions			
All participants (n = 41)	cws	p-long	np-long	*p* value
Walking speed (m / s)	0.83 ± 0.04	0.87 ± 0.05	0.85 ± 0.04	0.24
Cadence (step / min)	104.2 ± 2.95	98.2 ± 3.10	98.8 ± 2.95	< 0.001^a^^,^^b^
Symmetry index in single support phase (%)	-2.88 ± 0.55	-2.44 ± 0.49	-1.68 ± 0.48	0.21
Lower-limb peak flexion angle (deg)	13.1 ± 0.53	15.1 ± 0.51	14.0 ± 0.67	0.002^c^
Lower-limb peak extension angle (deg)	-18.1 ± 0.79	-19.1 ± 0.85	-21.8 ± 1.06	< 0.001^d^
VAF1	76.1 ± 0.95	74.1 ± 1.17	76.1 ± 0.89	0.17

The data are reported as the mean ± standard error. The *p* value indicates the result of repeated-measures one-way analysis of variance between walking conditions. The Bonferroni correction was used for post-hoc testing. Abbreviations: cws, comfortable walking speed; p-long, paralytic side long step; np-long, non-paralytic side long step; VAF, variance-accounted-for.

a) The results of the p-long condition were significantly lower than those of the cws condition (*p* < 0.01).

b) The results of the np-long condition were significantly lower than those of the cws condition (*p* < 0.05).

c) The results of the p-long condition were significantly higher than those of the cws condition (*p* < 0.001).

d) The results of the np-lng condition were significantly smaller than those of the cws and p-long conditions (*p* < 0.01).

The lower-limb flexion angle showed significant main effects for Condition (F (2, 68) = 6.03, *p* = 0.005, η_p_^2^ = 0.20) and for Subtype (F (2, 34) = 3.41, *p* = 0.050, η_p_^2^ = 0.22) and revealed that the interaction of Condition × Subtype was significant (F (4, 68) = 3.87, *p* = 0.008, η_p_^2^ = 0.24). Subtypes 2 (F (2) = 10.5, *p* < 0.001) and 3 (F (2) = 3.95, *p* = 0.04) revealed a significant simple main effect of Condition (Fig 2). The lower-limb extension angle showed significant main effects for Condition (F (2, 68) = 9.04, *p* < 0.001, η_p_^2^ = 0.26) and for Subtype (F (2, 34) = 19.5, *p* < 0.001, η_p_^2^ = 0.60) and revealed that the interaction of Condition × Subtype was significant (F (4, 68) = 3.22, *p* = 0.02, η_p_^2^ = 0.20). Further, the subtypes 2 (F (2) = 7.98, *p* = 0.003) and 3 (F (2) = 9.73, *p* = 0.002) revealed a significant simple main effect of Condition (Fig 2). In the post-hoc test, subtype 2 increased the lower-limb flexion angle in the np-long condition more than in the cws condition (17.5± 0.53 versus 14.5 ± 0.57, *p* = 0.007), and increased the lower-limb extension angle in the np-long condition more than in the cws (-26.1 ± 1.26 versus -19.4 ± 1.08, *p* < 0.001) and p-long conditions (-20.5 ± 1.51, *p* = 0.001). In subtype 3, the flexion angle of the lower-limb increased more in the p-long condition than in the cws condition (13.4 ± 0.56 versus 10.2 ± 0.55, *p* = 0.003), and the extension angle of the lower-limb increased more in the np-long condition than in the cws condition (-24.9 ± 0.70 versus -20.5 ± 1.28, *p* = 0.032). On the other hand, subtype 1 showed no difference in lower-limb flexion and extension angles among conditions (see S1 and S2 Tables for details).

### 3.3. Effect of experimental modulation of lower-limb angle on muscle synergies

We examined the effect of walking conditions on VAF1 (complexity of muscle synergy). First, VAF1 in the cws condition did not differ among the subtypes (*p* = 0.081).

**Fig 3:**
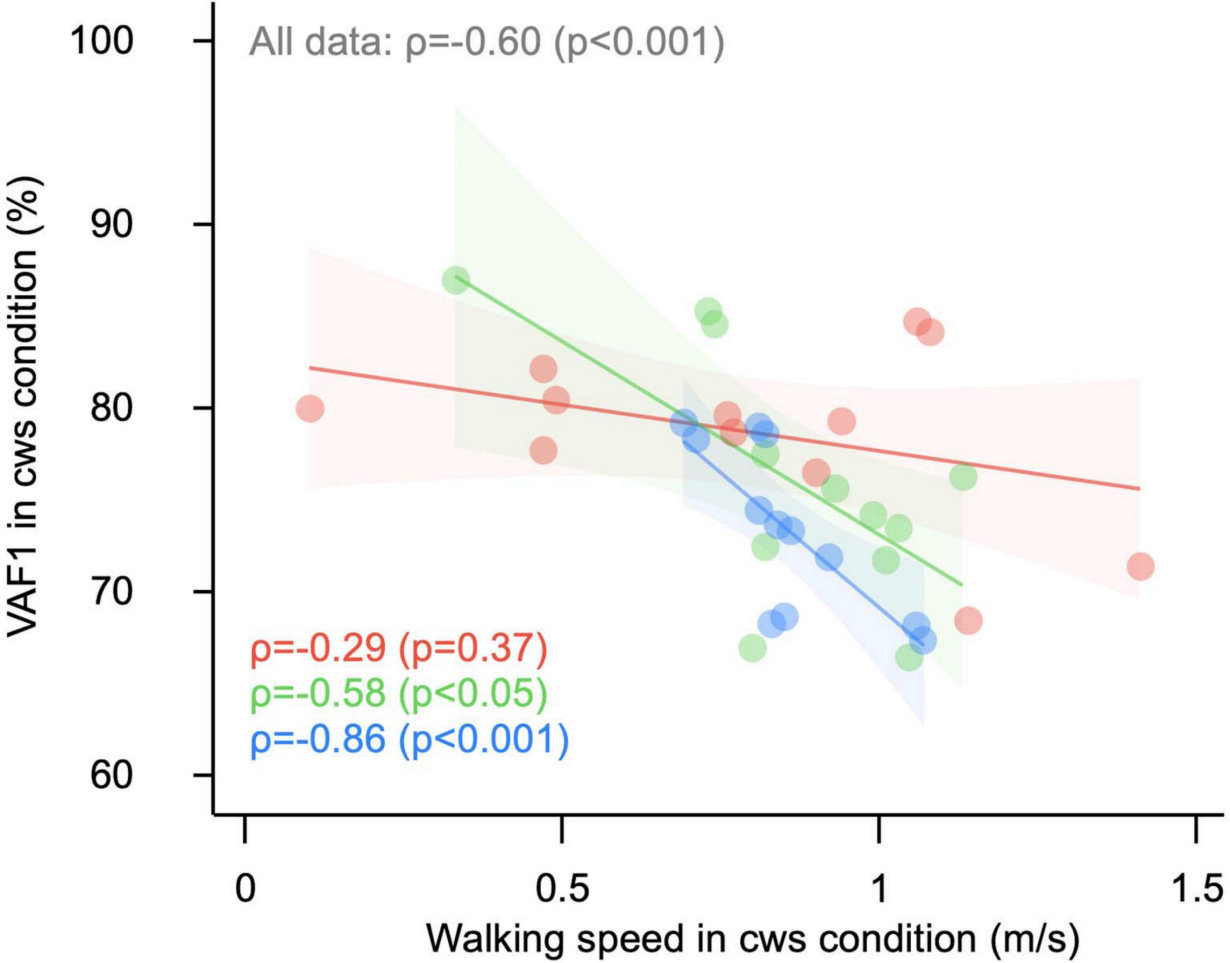
Association between walking speed and VAF1. The distribution shows the walking speed and VAF1 in each subtype. The line plot is a regression line and the shaded region indicates the 95% confidence interval. The top and right sides show the density plots for each subtype. Although walking speed and VAF1 showed a significant negative association, this was confirmed only for subtypes 2 and 3. Abbreviations: cws, comfortable walking speed; p-long, paralytic side long step; np-long, non-paralytic side long step; VAF, variance-accounted-for.

Next, we examined the effect of the walking condition on VAF1, and showed that VAF1 in all participants did not depend on walking condition (*p* = 0.174, Table 2), but the VAF1 did reveal condition dependencies when the sample was stratified by subtype. In addition, participants with greater walking speeds had smaller VAF1s, but this association was confirmed only for subtypes 2 (ρ = -0.58, *p* = 0.049) and 3 (ρ = -0.86, *p* < 0.001, see Fig 3).

VAF1 showed no main effect of Condition (F (1.61, 54.7) = 1.96, *p* = 0.16, η_p_^2^ = 0.06) in the RM-ANOVA with Greenhouse-Geisser correction. In contrast, VAF1 revealed a main effect of Subtype (F (2, 34) = 10.4, *p* < 0.001, η_p_^2^ = 0.39). The VAF1 revealed that the interaction of Condition × Subtype was significant (F (3.22, 54.7) = 2.59, *p* = 0.050, η_p_^2^ = 0.14). Because the interaction was significant, a simple main effect was confirmed. Subtype 3 only (F (2) = 4.29, *p* = 0.027) showed a significant simple main effect of Condition (Fig 2). Post-hoc test results revealed that VAF1 in subtype 3 was reduced in the p-long condition relative to the cws condition (68.0 ± 1.18 versus 73.5 ± 1.33, *p* = 0.036). However, in subtypes 1 (*p* = 0.133) and 2 (*p* = 0.550), VAF1 values were similar across walking conditions (see Table 3).

**Table 3:** Comparison of VAF1 across subtypes and walking conditions.

				95% Confidence Interval			
Walking conditions			Mean Difference	Lower	Upper	t value	p value
S1, cws	-	S2, cws	2.64	-4.23	9.51	1.28	1.00
	-	S3, cws	5.19	-1.69	12.1	2.51	0.52
	-	S1, p-long	-1.49	-6.86	3.88	-0.92	1.00
	-	S2, p-long	4.60	-2.27	11.5	2.22	1.00
	-	S3, p-long	10.7	3.85	17.6	5.18	< 0.001***
	-	S1, np-long	-1.23	-6.60	4.14	-0.77	1.00
	-	S2, np-long	4.49	-2.39	11.4	2.17	1.00
	-	S3, np-long	4.55	-2.33	11.4	2.20	1.00
S2, cws	-	S3, cws	2.55	-4.33	9.42	1.23	1.00
	-	S1, p-long	-4.12	-11.0	2.75	-1.99	1.00
	-	S2, p-long	1.96	-3.41	7.33	1.22	1.00
	-	S3, p-long	8.08	1.21	15.0	3.91	< 0.01**
	-	S1, np-long	-3.87	-10.7	3.00	-1.87	1.00
	-	S2, np-long	1.85	-3.52	7.22	1.15	1.00
	-	S3, np-long	1.91	-4.96	8.78	0.92	1.00
S3, cws	-	S1, p-long	-6.67	-13.5	0.20	-3.22	0.07
	-	S2, p-long	-0.58	-7.46	6.29	-0.28	1.00
	-	S3, p-long	5.54	0.17	10.9	3.44	0.04*
	-	S1, np-long	-6.42	-13.3	0.45	-3.10	0.10
	-	S2, np-long	-0.70	-7.57	6.17	-0.34	1.00
	-	S3, np-long	-0.64	-6.01	4.73	-0.40	1.00
S1, p-long	-	S2, p-long	6.09	-0.79	13.0	2.94	0.16
	-	S3, p-long	12.2	5.34	19.1	5.90	< 0.001***
	-	S1, np-long	0.25	-5.12	5.62	0.16	1.00
	-	S2, np-long	5.97	-0.90	12.8	2.89	0.18
	-	S3, np-long	6.03	-0.84	12.9	2.92	0.17
S2, p-long	-	S3, p-long	6.12	-0.75	13.0	2.96	0.15
	-	S1, np-long	-5.83	-12.7	1.04	-2.82	0.22
	-	S2, np-long	-0.12	-5.49	5.25	-0.07	1.00
	-	S3, np-long	-0.06	-6.93	6.82	-0.03	1.00
S3, p-long	-	S1, np-long	-12.0	-18.8	-5.08	-5.78	< 0.001***
	-	S2, np-long	-6.24	-13.1	0.64	-3.01	0.13
	-	S3, np-long	-6.18	-11.5	-0.81	-3.84	0.01*
S1, np-long	-	S2, np-long	5.72	-1.15	12.6	2.76	0.26
	-	S3, np-long	5.78	-1.09	12.6	2.79	0.24
S2, np-long	-	S3, np-long	0.06	-6.81	6.93	0.03	1.00

The *p* value indicates the result of repeated-measures two-way analysis of variance across subtypes and walking conditions. The Bonferroni correction was used for post-hoc testing. A high value indicates simplicity in the representation of muscle synergy. The effect of walking condition within the subtypes was only confirmed in subtype 3, and this subtype was reduced in the p-long condition compared to the cws condition. * *p* < 0.05,

** *p* < 0.01,

*** *p* < 0.001. Abbreviations: S1, Subtype 1; S2, Subtype 2; S3, Subtype 3; cws, comfortable walking speed; p-long, paralytic side long step; np-long, non-paralytic side long step; VAF, variance-accounted-for.

## 4. Discussion

The main purpose of this study was to identify the subtypes of muscle-synergy merging patterns during comfortable walking in subacute post-stroke patients and to determine the causal relationship of muscle synergy with experimentally modulated lower-limb flexion and extension angles during walking in this population. We found three subtypes of merging patterns and identified one subtype (merging of modules 3 and 4) that is distinct from the merging patterns reported in previous studies [13, 23]. Furthermore, only in subtype 3, the experimentally increased flexion angle of the paretic lower-limb during walking showed a more complex representation of muscle synergies. The findings suggest that the characteristic lower-limb flexion and extension angles observed during comfortable walking differ across subtypes, and that the effect of experimental modulation of the lower-limb angle during walking on the complexity of muscle synergy is different for each subtype.

### 4.1. Characteristics of comfortable walking and muscle-synergy merging in post-stroke patients

During comfortable walking, the decrease of lower-limb flexion angle or, conversely, the decrease of extension angle was determined for each participant (Fig 2). Furthermore, although only participants with the same three muscle synergies were analyzed in this study, the merging patterns varied among participants, and three subtypes were identified (Fig 1B) [13]. Subtype 1 was considered an impaired muscle synergy during stance [23], and subtype 2 was construed as an impaired muscle synergy during the transition from swing phase to stance [23]. In contrast, the current study identified a subtype 3, the merging of modules 3 and 4, which is interpreted as showing impaired muscle synergy during the swing phase in post-stroke patients. Previous studies have not discussed the pathological swing muscle synergy pattern in post-stroke patients; however, it has been demonstrated that this merged pattern is fractionated (i.e., re-separated) during the recovery process [49]. However, since we did not show the fractionation index of muscle synergy, the involvement of such a mechanism is unclear. In the cws condition, the subtypes varied in the peak limb-movement angles observed, with subtypes 1 showing reduced lower-limb extension angles, while subtype 3 had decreased lower-limb flexion angles. Since the neural system controls the basic pattern of limb movements during walking [32, 33], we believe that each subtype reflects a different neural pathology. Subtype 3 reflects the merging of modules 3 and 4 (Fig 1B), which suggests that the activity of module 4 in the first half of the swing phase inhibited hip flexion and knee extension movements [50]. As a result, a feature of subtype 3 is believed to be restriction of the limb flexion angle in the cws condition by the early activation of module 4. Corticospinal damage is associated with decreased hip flexion angle during walking [51]. In healthy subjects, activation of the hamstrings (e.g., the biceps femoris) during the swing phase is associated with corticospinal tract excitability [52], suggesting that early activation of the hamstrings occurs in subtype 3 as a result of corticospinal dysfunction. Nevertheless, since we did not measure corticospinal tract excitability, this is only a speculative assumption.

### 4.2. Effect of experimental modulation of lower-limb angle on muscle synergy

The lower-limb flexion angle increased in the p-long condition, and the lower-limb extension angle increased in the np-long condition in all participants. Our experimental design manipulated the kinematic parameters without altering walking speed. Although the VAF1 did not differ across walking conditions in subtypes 1 and 2, subtype 3 showed a reduction under the p-long condition (Fig 2). Since the VAF1 did not vary with walking condition across all participants, we believe that the observation of a decrease in VAF1 with experimental limb angle modulation depended on the subtype during comfortable walking. Because subtype 3 had a decreased lower-limb flexion angle in the cws condition, we believe that VAF1 was reduced (i.e., a greater complexity) when the required lower-limb swing increased in the p-long condition. The finding that the number of muscle synergies increased when participants were required to perform under difficult walking conditions [12] is consistent with our results that muscle synergy complexity in subtype 3 increased with lower-limb flexion angle (p-long condition). However, it has also been reported that the number of muscle synergies do not change by altering the step height or stride length during walking [53]. Most studies examining muscle synergy during walking following a stroke have included patients in the chronic phase [13, 15, 23–25], and our results may be specific only to the subacute post-stroke patients involved in our study. We believe that this was due to the flexibility of motor patterns and central nervous system activity in subacute patients compared to those in the chronic phase [54–56]. Furthermore, although the p-long and n-plong conditions required participants to voluntarily alter the lower-limb angle, subtype 1 experienced more severe motor paresis than the other subtypes, and the voluntary control of lower-limb movements was considered more difficult in subtype 1. Therefore, VAF1 might be altered on a split-belt treadmill that did not require the modulation of a voluntary lower-limb angle [57].

Although previous studies have assessed the complexity of muscle synergy by the number of muscle synergies [13, 15, 23, 49], we used VAF1 as a measure of the complexity of muscle synergy. Because the number of muscle synergies was determined based on the value of VAF [12–15], the number of muscle synergies and VAF1 are essentially similar. However, because the threshold for determining the number of muscle synergies is an arbitrary value, VAF1 is likely to be more sensitive in capturing the complexity of muscle synergies than the number of muscle synergies [12, 16–20].

### 4.3. Limitations and future directions

This study has some limitations. 1) We did not investigate the possibility that the state of the non-paretic side affected the paretic side because the electromyogram was recorded only on the paretic side. 2) We did not investigate whether the effects of the p-long and np-long conditions on VAF1 were neurological phenomena specific to post-stroke patients because we did not have a healthy control group. Analysis of the brain lesions giving rise to subtype 3 is necessary to clarify the relationship between early activation of the hamstrings in the cws condition and corticospinal damage. Future studies are also needed to provide insight into the mechanism of VAF1 reduction by the p-long condition in subtype 3. Nevertheless, this study identified post-stroke patients with merged muscle synergies involving modules 3 and 4 and showed that this subtype has a reduced lower-limb flexion angle during comfortable walking. In subtype 3 only, muscle synergy was represented in a more complex manner when the lower-limb flexion angle during walking was experimentally increased. The detailed subtype classification based on the merging pattern of muscle synergies will be useful for selecting rehabilitation appropriate to the specific neurological disorder of each patient. In walking training for patients with merging of the swing-muscle synergies, rehabilitation promoting an increased lower-limb flexion angle may render the muscle synergies more complex.

## 5. Conclusion

Our findings identified three impairment subtypes based on the merging pattern of the muscle synergies observed during comfortable walking, including a new subtype consisting of the merging of modules 3 and 4, in which the lower-limb flexion angle was decreased during comfortable walking. However, walking with a larger lower-limb flexion angle in this subtype resulted in a more complex representation of muscle synergies. Thus, a detailed subtype classification based on the merging pattern of muscle synergies could be useful for selecting walking rehabilitation interventions targeted to the specific neurological disorder of each patient.

## Supporting information

S1 Table Comparison of lower-limb peak flexion angles across subtypes and walking conditions. The *p* value indicates the result of repeated-measures two-way analysis of variance across subtypes and walking conditions. The Bonferroni correction was used for post-hoc testing. The high value of lower-limb angle was set as the direction of flexion. Subtype 3 was reduced more than other subtypes in the cws condition. Subtype 3 was significantly increased in the p-long condition compared to the cws condition. * *p* < 0.05, ** *p* < 0.01, *** *p* < 0.001. Abbreviations: S1, Subtype 1; S2, Subtype 2; S3, Subtype 3; cws, comfortable walking speed; p-long, paralytic side long step; np-long, non-paralytic side long step.
(DOCX)

S2 Table Comparison of lower-limb peak extension angles across subtypes and walking conditions. The *p* value indicates the result of repeated-measures two-way analysis of variance across subtypes and walking conditions. The Bonferroni correction was used for post-hoc testing. The high value of lower-limb angle was set as the direction of flexion. Subtype 1 was reduced compared to the other subtypes in the cws condition. Subtypes 2 and 3 were significantly decreased in the np-long condition compared to the cws condition. In contrast, subtype 1 did not differ across walking conditions. * *p* < 0.05, ** *p* < 0.01, *** *p* < 0.001. Abbreviations: S1, Subtype 1; S2, Subtype 2; S3, Subtype 3; cws, comfortable walking speed; p-long, paralytic side long step; np-long, non-paralytic side long step.
(DOCX)
