# Supplementary material for: Merged swing-muscle synergies and their relation to walking characteristics in subacute post-stroke patients: An observational study
Source: PLoS One. 2022 Feb 4;17(2):e0263613. doi: 10.1371/journal.pone.0263613 (PMC8815905; doi:10.1371/journal.pone.0263613)
Supplement: S1 Table — The p value indicates the result of repeated-measures two-way analysis of variance across subtypes and walking conditions. The Bonferroni correction was used for post-hoc testing. The high value of lower-limb angle was set as the direction of flexion. Subtype 3 was reduced more than other subtypes in the cws condition. Subtype 3 was significantly increased in the p-long condition compared to the cws condition. * p < 0.05, ** p < 0.01, *** p < 0.001. Abbreviations: S1, Subtype 1; S2, Subtype 2; S3, Subtype 3; cws, comfortable walking speed; p-long, paralytic side long step; np-long, non-paralytic side long step. (DOCX) [file pone.0263613.s001.docx]

**Supporting information**

**S1 Table. Comparison of lower-limb peak flexion angles across subtypes and each walking condition**

|  |  |  |  | 95% Confidence Interval | |  |  |
| --- | --- | --- | --- | --- | --- | --- | --- |
| Walking conditions | | | Mean Difference | Lower | Upper | t value | p value |
| S1, cws | - | S2, cws | 0.04 | -3.87 | 3.96 | 0.04 | 1.00 |
|  | - | S3, cws | 4.32 | 0.41 | 8.23 | 3.70 | 0.02* |
|  | - | S1, p-long | -1.70 | -4.26 | 0.85 | -2.23 | 1.00 |
|  | - | S2, p-long | -1.18 | -5.10 | 2.73 | -1.01 | 1.00 |
|  | - | S3, p-long | 1.10 | -2.81 | 5.01 | 0.94 | 1.00 |
|  | - | S1, np-long | 1.60 | -0.96 | 4.15 | 2.09 | 1.00 |
|  | - | S2, np-long | -2.97 | -6.88 | 0.94 | -2.55 | 0.48 |
|  | - | S3, np-long | 2.95 | -0.96 | 6.86 | 2.53 | 0.51 |
| S2, cws | - | S3, cws | 4.28 | 0.37 | 8.19 | 3.67 | 0.02* |
|  | - | S1, p-long | -1.75 | -5.66 | 2.17 | -1.50 | 1.00 |
|  | - | S2, p-long | -1.23 | -3.78 | 1.33 | -1.60 | 1.00 |
|  | - | S3, p-long | 1.06 | -2.85 | 4.97 | 0.91 | 1.00 |
|  | - | S1, np-long | 1.56 | -2.36 | 5.47 | 1.33 | 1.00 |
|  | - | S2, np-long | -3.02 | -5.57 | -0.46 | -3.94 | < 0.01** |
|  | - | S3, np-long | 2.91 | -1.01 | 6.82 | 2.49 | 0.56 |
| S3, cws | - | S1, p-long | -6.03 | -9.94 | -2.11 | -5.17 | < 0.001*** |
|  | - | S2, p-long | -5.51 | -9.42 | -1.59 | -4.72 | < 0.001*** |
|  | - | S3, p-long | -3.22 | -5.78 | -0.66 | -4.21 | < 0.01** |
|  | - | S1, np-long | -2.72 | -6.64 | 1.19 | -2.33 | 0.83 |
|  | - | S2, np-long | -7.29 | -11.2 | -3.38 | -6.25 | < 0.001*** |
|  | - | S3, np-long | -1.37 | -3.93 | 1.18 | -1.79 | 1.00 |
| S1, p-long | - | S2, p-long | 0.52 | -3.39 | 4.43 | 0.45 | 1.00 |
|  | - | S3, p-long | 2.81 | -1.11 | 6.72 | 2.41 | 0.69 |
|  | - | S1, np-long | 3.30 | 0.75 | 5.86 | 4.31 | < 0.01** |
|  | - | S2, np-long | -1.27 | -5.18 | 2.64 | -1.09 | 1.00 |
|  | - | S3, np-long | 4.65 | 0.74 | 8.56 | 3.99 | < 0.01** |
| S2, p-long | - | S3, p-long | 2.29 | -1.63 | 6.20 | 1.96 | 1.00 |
|  | - | S1, np-long | 2.78 | -1.13 | 6.69 | 2.38 | 0.73 |
|  | - | S2, np-long | -1.79 | -4.35 | 0.77 | -2.34 | 0.81 |
|  | - | S3, np-long | 4.13 | 0.22 | 8.04 | 3.54 | 0.03* |
| S3, p-long | - | S1, np-long | 0.50 | -3.42 | 4.41 | 0.43 | 1.00 |
|  | - | S2, np-long | -4.07 | -7.99 | -0.16 | -3.49 | 0.03* |
|  | - | S3, np-long | 1.85 | -0.71 | 4.40 | 2.41 | 0.67 |
| S1, np-long | - | S2, np-long | -4.57 | -8.48 | -0.66 | -3.92 | < 0.01** |
|  | - | S3, np-long | 1.35 | -2.56 | 5.26 | 1.16 | 1.00 |
| S2, np-long | - | S3, np-long | 5.92 | 2.01 | 9.83 | 5.07 | < 0.001*** |

The p value indicates the result of repeated-measures two-way analysis of variance across subtypes and each walking condition. The Bonferroni correction was used for post-hoc testing. The high value of lower-limb angle is set as the direction of flexion. Subtype 3 was reduced more than other subtypes in the cws condition. Subtype 3 was significantly increased in the p-long condition compared to the cws condition. * p < 0.05, ** p < 0.01, *** p < 0.001. Abbreviations: S1, Subtype 1; S2, Subtype 2; S3, Subtype 3; cws, comfortable walking speed; p-long, paralytic side long step; np-long, non-paralytic side long step.
