# Supplementary material for: Merged swing-muscle synergies and their relation to walking characteristics in subacute post-stroke patients: An observational study
Source: PLoS One. 2022 Feb 4;17(2):e0263613. doi: 10.1371/journal.pone.0263613 (PMC8815905; doi:10.1371/journal.pone.0263613)
Supplement: S2 Table — The p value indicates the result of repeated-measures two-way analysis of variance across subtypes and walking conditions. The Bonferroni correction was used for post-hoc testing. The high value of lower-limb angle was set as the direction of flexion. Subtype 1 was reduced compared to the other subtypes in the cws condition. Subtypes 2 and 3 were significantly decreased in the np-long condition compared to the cws condition. In contrast, subtype 1 did not differ across walking conditions. * p < 0.05, ** p < 0.01, *** p < 0.001. Abbreviations: S1, Subtype 1; S2, Subtype 2; S3, Subtype 3; cws, comfortable walking speed; p-long, paralytic side long step; np-long, non-paralytic side long step. (DOCX) [file pone.0263613.s002.docx]

**Supporting information**

**S2 Table. Comparison of lower-limb peak extension angles across subtypes and each walking condition**

|  |  |  |  | 95% Confidence Interval | |  |  |
| --- | --- | --- | --- | --- | --- | --- | --- |
| Walking conditions | | | Mean Difference | Lower | Upper | t value | p value |
| S1, cws | - | S2, cws | 4.97 | -0.24 | 10.2 | 3.16 | 0.08 |
|  | - | S3, cws | 6.11 | 0.90 | 11.3 | 3.89 | < 0.01** |
|  | - | S1, p-long | 0.14 | -4.00 | 4.28 | 0.11 | 1.00 |
|  | - | S2, p-long | 6.07 | 0.86 | 11.3 | 3.87 | < 0.01** |
|  | - | S3, p-long | 7.85 | 2.64 | 13.1 | 5.00 | < 0.001*** |
|  | - | S1, np-long | -0.13 | -4.27 | 4.02 | -0.10 | 1.00 |
|  | - | S2, np-long | 11.6 | 6.42 | 16.8 | 7.41 | < 0.001*** |
|  | - | S3, np-long | 10.4 | 5.21 | 15.6 | 6.64 | < 0.001*** |
| S2, cws | - | S3, cws | 1.14 | -4.07 | 6.35 | 0.72 | 1.00 |
|  | - | S1, p-long | -4.83 | -10.0 | 0.38 | -3.07 | 0.11 |
|  | - | S2, p-long | 1.11 | -3.04 | 5.25 | 0.89 | 1.00 |
|  | - | S3, p-long | 2.88 | -2.33 | 8.09 | 1.83 | 1.00 |
|  | - | S1, np-long | -5.09 | -10.3 | 0.12 | -3.24 | 0.06 |
|  | - | S2, np-long | 6.67 | 2.52 | 10.8 | 5.37 | < 0.001*** |
|  | - | S3, np-long | 5.46 | 0.25 | 10.7 | 3.47 | 0.03* |
| S3, cws | - | S1, p-long | -5.96 | -11.2 | -0.75 | -3.80 | 0.01* |
|  | - | S2, p-long | -0.03 | -5.24 | 5.18 | -0.02 | 1.00 |
|  | - | S3, p-long | 1.74 | -2.40 | 5.88 | 1.40 | 1.00 |
|  | - | S1, np-long | -6.23 | -11.4 | -1.02 | -3.97 | < 0.01** |
|  | - | S2, np-long | 5.53 | 0.32 | 10.7 | 3.52 | 0.03* |
|  | - | S3, np-long | 4.32 | 0.18 | 8.46 | 3.48 | 0.03* |
| S1, p-long | - | S2, p-long | 5.93 | 0.72 | 11.1 | 3.78 | 0.01* |
|  | - | S3, p-long | 7.71 | 2.50 | 12.9 | 4.91 | < 0.001*** |
|  | - | S1, np-long | -0.27 | -4.41 | 3.88 | -0.21 | 1.00 |
|  | - | S2, np-long | 11.5 | 6.28 | 16.7 | 7.32 | < 0.001*** |
|  | - | S3, np-long | 10.3 | 5.07 | 15.5 | 6.55 | < 0.001*** |
| S2, p-long | - | S3, p-long | 1.77 | -3.44 | 6.98 | 1.13 | 1.00 |
|  | - | S1, np-long | -6.20 | -11.4 | -0.99 | -3.95 | < 0.01** |
|  | - | S2, np-long | 5.56 | 1.42 | 9.70 | 4.48 | < 0.01** |
|  | - | S3, np-long | 4.35 | -0.86 | 9.56 | 2.77 | 0.25 |
| S3, p-long | - | S1, np-long | -7.97 | -13.2 | -2.76 | -5.08 | < 0.001*** |
|  | - | S2, np-long | 3.79 | -1.42 | 9.00 | 2.41 | 0.66 |
|  | - | S3, np-long | 2.58 | -1.57 | 6.72 | 2.08 | 1.00 |
| S1, np-long | - | S2, np-long | 11.8 | 6.55 | 17.0 | 7.49 | < 0.001*** |
|  | - | S3, np-long | 10.5 | 5.34 | 15.8 | 6.72 | < 0.001*** |
| S2, np-long | - | S3, np-long | -1.21 | -6.42 | 4.00 | -0.77 | 1.00 |

The p value indicates the result of repeated-measures two-way analysis of variance across subtypes and each walking condition. The Bonferroni correction was used for post-hoc testing. The high value of lower-limb angle is set as the direction of flexion. Subtype 1 was reduced compared to the other subtypes in the cws condition. Subtypes 2 and 3 were significantly decreased in the np-long condition comparing to the cws condition. In contrast, subtype 1 did not differ across walking conditions. * p < 0.05, ** p < 0.01, *** p < 0.001. Abbreviations: S1, Subtype 1; S2, Subtype 2; S3, Subtype 3; cws, comfortable walking speed; p-long, paralytic side long step; np-long, non-paralytic side long step.
